# Remedies and Their Uses

**Published:** 1907-04-20

**Authors:** 


### Remedies and their Uses.

### Drugs acting on the Bronchial Mucosa.

*Pyrenol.*—This body is a compound of salicylic acid, benzoic acid, and thymol, and is a white crystalline powder, slightly hygroscopic and possessing an aromatic odour and slightly burning taste. It produces a mild diaphoresis and is a cardiac tonic. It has no irritant action on the digestive mucosa. It has been found valuable in pneumonia, where its stimulant action on the heart is of importance (Winterberg, Wein. Klin. Rundsch., 1905). A number of cases of bronchial asthma have also been benefited, as it promotes expectoration and acts as a sedative to the sensory nerve endings. From its composition it will be seen to possess antiseptic properties, and thus it has been found useful in chronic bronchitis with fœtor and pyrexia. In pertussis one observer has recorded good results. It is not apparently of much value in tuberculosis. The dose is 5 to 15 grains three to five times daily, either in tablets or in cold water, milk, tea, coffee, or cocoa. It is decomposed by heat. It may also be given in powders. In a mixture the solution (5 per cent. for adults or 3 per cent. for children) may be combined with oleum menth. pip., to disguise the taste, and, if necessary, syr. codeinæ or heroine hydrochloride may be added to the mixture. It is supplied by Reitmeyer and Co.

*Tussol* is the mandelic acid ester of antipyrin, and is a white crystalline powder, soluble in 15 parts of water, and much more soluble in alcohol. It is decomposed by alkalies and also by milk, so that it should never be prescribed with these substances or shortly before or after a milk meal. It possesses the antipyretic and analgesic action of antipyrin, and is also a stimulant to the secretory glands. It is indicated in bronchial and laryngeal catarrh, but especially in pertussis, for which condition antipyrin itself has often been found beneficial. No ill-effects have been observed after its use in children, and some observers consider that it is superior to antipyrin, and distinctly shortens the duration of the attack in whooping-cough. The dosage is:—

Children under 1 year 2-5 grains per diem in divided doses.
2 to 3 years 5-10 grains per diem.
3 to 5 years 15-20 grains per diem.

In older children and adults 7½ grains may be given three or more times daily. It may be made up with syrupus aurant. or any other convenient flavouring agent. It is prepared by Meister, Lucius, and Brüning.

*Grindelia robusta.*—The liquid extract of grindelia of the British Pharmaceutical Conference is made with alcohol; Messrs. Parke, Davis now prepare an extract in which an aqueous alkaline menstruum is used, which is said to secure a more efficient pharmacological action, and also has the advantage of compatibility with syrups and aqueous fluids without precipitation. It has a sedative action on the sensory and motor nerve endings, and in large doses the central nervous system also becomes affected. Eventually a narcotic or comatose condition is produced. In small and moderate medicinal doses the heart is quickened and the blood pressure raised; in large doses there is slowing of the heart, vaso-dilatation and fall in blood pressure. It is owing to its effect on the vessels that its diuretic action appears mainly to be due. Its action on the sensory nerve endings in the respiratory mucosa and its stimulating effect in small doses on the respiration suggest its value in asthma and bronchial catarrh; recent investigations have failed to determine which of its constituents is the active principle, and have given discordant results as to the nature of these bodies. In the present state of our knowledge, therefore, it is of importance to employ some standard preparation which can be relied on to contain all the ingredients of the plant. The following prescriptions have been advised in asthmatic conditions:—

| | |
|---|---|
| Ext. grindeliæ fluid. ... ... ... | ℥ij. |
| Pot. iod. ... ... ... ... ... ... | ʒij. |
| Syr. tolutani ad ... ... ... ... | ℥iv. |

F.M. One teaspoonful every 3 hours.

| | |
|---|---|
| Ext. grindeliæ fluid. ... ... ... | ℥iv. |
| Syr. rhei | |
| Syr. Sennæ ... ... ... | aa. ℥j. |

F.M. S.: A dessertspoonful (ʒiij.) every half hour during the spasm; when relieved the same dose every 4 hours.

*Drosera.*—A fluid extract of this plant, the well-known "Sundew," is also prepared by Parke, Davis and Co., as an expectorant especially in whooping-cough. Although the reports are not all favourable to its action in the latter complaint, it appears to have been efficacious in many instances, while it appears in therapeutic doses to have no ill effects of a secondary nature. The fluid extract is not miscible with water; the dose is 5 to 20 minims for adults, and less for children.

*Saw Palmetto.*—This is another drug prepared by the same firm which has recently been recommended in bronchial and laryngeal affections as a sedative. It is also credited with a general stimulating action on metabolism. In acute laryngitis and in whooping-cough it has met with some success. The dose of the fluid extract, which is not miscible with water, is ½ to 2 drachms.

*Pertussin* is a fluid extract of German thyme made up to a strength of 1 in 7 with syrup. There is no other active ingredient. As its trade name implies, it is intended specially as a specific for whooping-cough, but its action, which is apparently that of a disinfectant expectorant, will enable it to be used in acute and chronic bronchitis and other affections of the respiratory passages. It is miscible with alcohol or milk. In whooping-cough ½ to 1 drachm doses are given frequently, but in older patients half an ounce may be ordered thrice daily or oftener. It appears to have no ill after-effects. It is obtainable in England from Messrs. Christy and Co.

